# Maternal performance after childbirth and its predictors: a cross sectional study

**DOI:** 10.1186/s12884-024-06412-3

**Published:** 2024-03-22

**Authors:** Masoumeh Choobdarnezhad, Leila Amiri-Farahani, Sally Pezaro

**Affiliations:** 1 Department of Reproductive Health and Midwifery, School of Nursing and Midwifery, Iran University of Medical Sciences, Tehran, Iran; 2 Department of Reproductive Health and Midwifery, Nursing and Midwifery Care Research Center, School of Nursing and Midwifery, Iran University of Medical Sciences, Tehran, 1996713883 Iran; 3 The Research Centre for Healthcare and Communities, Coventry University, Coventry, UK; 4 The University of Notre Dame, Notre Dame, Fremantle, Australia

**Keywords:** Birth experience, Maternal performance, Maternal self-efficacy, Predictors, Postpartum

## Abstract

**Background and Objectives:**

Birthing parents need to use specialized skills as the first caregiver of the newborn. Several factors may affect performance. Yet there is a paucity of research in this area, and evidence remains inconsistent. Consequently, this study aimed to determine maternal performance after childbirth and its predictors.

**Methods:**

This cross-sectional study was conducted with those (*n* = 450) who had given birth (< two months) and been referred for the vaccination of their newborn. The multi-stage sampling method was carried out from April 2022 to February 2023. Participants who met the inclusion criteria completed a demographic and obstetric information questionnaire, along with the childbirth experience 2 (CEQ2), Barkin maternal performance and maternal self-efficacy scales. Multiple linear regression was used to investigate the predictive effect of the independent variables of childbirth experience, maternal self-efficacy, demographic and obstetric variables on the dependent variable of maternal performance.

**Results:**

The mean age of the participants was 26.78 and the mean total score of maternal performance was 91.04 (0—120). The highest and lowest scores related to the ‘maternal competence’ and the ‘maternal needs’ domains, with mean score calculated at 77.51 and 72.81 respectively. ‘Childbirth experience’ and ‘maternal self-efficacy’ domains had a statistically significant relationship with maternal performance (*P* < 0.05). Among the predictive factors of maternal performance, the results of our linear regression demonstrated the variables of birth experience (B = 0.63), maternal self-efficacy (B = 1.53), spouse's employment status (B = 5.78 for worker level, B = 3.99 for employee level), the number of previous childbirth experiences (B = -8.46), frequency of receiving antenatal care (B = -6.68), length of stay in the birth suite (B = -2.22) and length of stay in the hospital (B = 2.84) remained in the model. 53.2% of changes in maternal performance can be explained by these independent variables.

**Conclusion:**

The promotion of evidence-based, person-centered, and respectful perinatal care during pregnancy and childbirth are of paramount importance. Strategies to improve the experience of childbirth and self-efficacy are especially required to improve maternal performance in the postpartum period. Prenatal care aimed at improving maternal function after childbirth will be important in achieving this overall.

## Introduction

Giving birth is one of the most important experiences in life for many [1]. The birth experience is often considered a positive and natural event and can have many effects on both the health of the birthing person and the neonate [2]. A positive birth experience is associated with increased bonding and a higher rate of breastfeeding. It is also known to increase patience, responsibility, and independence in parents who have just given birth [3, 4]. Contrariwise, a negative birth experience can be harmful and even damaging in some cases [5], causing disturbances in the development of the neonate, nutrition, sleep and even mood in early childhood [6]. For the birthing parent, it can cause a decrease in future fertility, increased rates of abortion [7] and increased requests for cesarean birth in subsequent pregnancies [8]. Negative childbirth experiences also increase rates of postpartum depression, post-traumatic stress disorder and negative feelings towards the child [7]. As such, the ways in which to promote more positive childbirth experiences must be wholly understood and prioritized.

In Iran, disrespectful behaviour towards birthing parents has a relatively high prevalence, particularly where birth occurs in government centres and is facilitated by medical students or on call physicians [9, 10]. Childbirth, rather than being managed as a physiological event has become increasingly medicalized in Iran, and many choose a medical model of care, despite their inclination toward physiological childbirth. Undoubtedly, medical approaches are necessary in specific circumstances, but overexpansion of medicalization can interfere with outcomes and birth choices. In previous studies, we have tried to provide a broad description of the medicalization of childbirth in Iran via qualitative inquiry [11]. Conversely, health authorities can prevent the adverse effects associated with birth being medicalized and physiological childbirth through evidence-based care [10]. When physiological childbirth is pathologized via medical approaches, the midwife’s role as expert in the process of childbirth changes [12]. More importantly, medicalization changes people’s perceptions of midwives’ professional skills; therefore, obstetricians (medical experts in pathology rather than physiology) often replace midwives in leading perinatal care [13].

The prevalence of negative childbirth experiences has been reported in different parts of the world from 4.6 to 44% [7]. Scores related to birth experience in Iran have been reported at 59.08 (21–84) [14], demonstrating a tendency for birth experience scores to be relatively high and thus negative [15]. The prevalence of traumatic childbirth in Iran has also been reported at 3.48% [4]. Collectively, this suggests that the prevalence of negative birth experiences in Iran may be reasonably high. Yet further research will be required to wholly understand this phenomenon in the context of Iran.

Birth experience has many effects on the postpartum period, particularly upon maternal performance, which is one of the important indicators in the successful transition of role and is predictive of behavior, self-efficacy and approach to infant care [16]. Postpartum maternal functioning encompasses various dimensions, including personal care, child and family care, and social and occupational activities [17]. Maternal functioning can improve substantially between the first and sixth week of the postpartum period; however, a number of birthing parents take up to six months to achieve a desirable level of functioning [18]. Maternal performance is also influenced by age and parity [19], financial resources, communication skills, physical environment, self-care, and social support offered by the family [20]. Havizari et al. reported that mode of birth; birth experience and self-care are also related factors impacting upon maternal performance [21]. Satisfaction with one’s birth experience and having a positive birth experience also increases the self-efficacy score post-birth [7]. Yet it will be important to further understand and determine maternal performance after childbirth and its predictors in the context of Iran specifically, where little is known on this topic, and the prevalence of negative birth experiences may be high.

In England, those who had better support during labor and a positive birth experience displayed more favorable infant care behaviors following childbirth [22]. Contrariwise, postpartum depression, being a first child, having a low family income and being primiparous have been associated with a weaker level of maternal performance, where there is also a significant relationship between self-efficacy and the total score of maternal performance [23]. While one study reported younger participants to have higher self-efficacy, there was also a reported relationship between the level of education and self-efficacy, where those with a higher level of education had a higher self-efficacy score [24]. Age may not be related to maternal performance [25]. Yet those with a higher literacy level have reportedly had better maternal performance after childbirth [19]. This conflicting evidence is indicative of a need for further research, particularly in geographical areas where less research has been conducted thus far, and yet levels of negative childbirth experiences may be high.

Despite the contradictions regarding factors related to maternal performance, the Inventory of Functional Status After Childbirth tool (IFSAC) has predominantly been used to measure maternal performance in previous studies [26]. The use of more accurate tools to measure maternal performance may enhance the quality of evidence. Equally, in one study, samples were selected only from Tabriz city, urban areas and included only those with low-risk pregnancies whilst excluding those from marginalized and rural communities with high-risk pregnancies. As such, there is a need to include participants from communities where the Azerbaijani Turk culture is less dominent (e.g., in Tabriz city, Iran) [21] and with more ethnically diverse community groups to enable any evidence genderated to be more generalisable. Taking into account the limitations of previous studies, this research was designed to include participants with high-risk, as well as low risk pregnancies, use Barkin's maternal performance measurement tool, employ multi-stage sampling, and sampling from the mega city of Tehran, where people with a broader diversity of cultures and ethnicities live. Considering the above, the overall objective of this study was to determine maternal performance after childbirth and its predictors. Specific aims included 1) determining the mean score of maternal performance, childbirth experience and maternal self-efficacy, 2) comparing maternal performance, childbirth experience and maternal self-efficacy between high risk pregnancy (HRP) and low risk prgancy (LRP), and 3) determining the relationship between childbirth experience and maternal self-efficacy, demographics and fertility with maternal performance, and 3) determining the predictors of maternal performance.

## Methods

### Study design

The current cross-sectional study includes participants who were referred for neonatal vaccinations via the health centers affiliated with the University of Medical Sciences, Tehran, Iran, two-months after giving birth. In order to select participants, multi-stage sampling was used. Firstly, health centers affiliated to the university were divided into two strata, (west and northwest). After random selection of centers from both west and north-west strata, sampling from each center was done using the proportional allocation method. Continuous sampling was used until the full sample size was reached among those who met the inclusion criteria. The share of participants recruited to the study was determined by the total number of people who gave birth and were referred to each of the West and Northwest health centers equally. Information about the questionnaire was given to potential participants by one of the authors (M.CH.), who were then invited to offer their consent and complete the instruments in paper form. Sampling occurred from May 2022 to February 2023.

### Sample size

Sample size has been calculated previously in similar studies published elsewhere at 246 [19], 385 [23], and 385 people [21]. After reviewing the volume of different samples obtained in these previous studies whilst anticipating an approximate 10% dropout rate, the highest sample volume was considered equal to 450. Confidence limits of 95% and a power of 80% were also considered in these calculations.

### Inclusion and exclusion criteria

All participants who met the inclusion criteria were Iranian, and had vaginal births, singleton pregnancies, and either low-risk or high-risk pregnancies (e.g., due to diabetes, anemia, hypothyroidism, blood pressure, preeclampsia, a body mass index above 29, age ≥ 35 or ≤ 18, low birth weight and/or premature birth). Those who were not Iranian or had experienced stressful events such as the death of a loved one in the last three months; an instrumental vaginal birth; babies born with abnormalities; a history of alcohol, smoking and/or drug use; known psychological conditions, or underlying physical conditions (e.g., lupus, heart disease, and kidney disease) were excluded from participation.

### Data collection

The first part of the data collection tool included a demographic and obstetric history questionnaire. The second part of the data collection tool contained the Childbirth Experience Questionnaire (CEQ). This questionnaire was designed by Dencker et al., and includes 23 items, 20 of which are scored with a 4-point Likert scale (completely agree = 4, to completely disagree = 1). The other three items include remembering the pain of childbirth, sense of security and control and are scored using a visual analog scale 0–100 (score 25–40 = 1, score 41–60 = 2, score 61–80 = 3, score 81–100 = 4). Items with reverse scoring are also included: 3, 5, 8, 13, 14, 19, 20, 21 (8 items). Childbirth experience is measured in four domains, ‘participation’ (8, 9, 10, 12), ‘own capacity’ (1, 2, 4, 5, 6, 7, 21, 22), ‘professional support’ (11, 13, 14, 15, 16) and ‘perceived safety’ (3, 17, 18, 19, 20, 23). The instrument’s validity is based on face and content validity. Reliability has been confirmed through internal consistency with Cronbach’s alpha 0.70, as the score increases, so does the level of positive birth experience [15]. The validity and reliability of the Iranian version has also been confirmed and previously published [27].

The third section of the data collection tool contained the maternal self-efficacy scale, which has been designed by Gefand and Teti and includes 10 items [28]. Each item is scored on a 4-point Likert scale (very worse = 1, to better than others = 4). A higher score is associated with better self-efficacy. The face and content validity of this tool has been confirmed, and its reliability has been reported with Cronbach’s alpha internal consistency of 86% [28]. The validity and reliability of the Iranian version of the tool has also been confirmed and published elsewhere [29].

The fourth part of the data collection tool included the Barkin index of maternal functioning (BIMF) instrument [17] which includes 20 items. Each item is scored on a 7-point Likert scale (strongly disagree = 0 to strongly agree = 6). The minimum score is zero and the maximum score is 120. A higher score is associated with a higher level of maternal performance. This tool has two domains, the domain of ‘maternal needs’ includes 7 items (2, 6, 7, 8, 9, 11 and 13) and the domain of ‘maternal competence’ includes 13 items (1, 3, 4, 5, 10, 12, 14, 15, 16, 17, 18, 19 and 20). In order to complete the questionnaire, the participant is asked to indicate their experience of maternal performance during the previous two weeks. Items 16 and 18 have reverse scoring. In the original version of the instrument, its reliability was confirmed with Cronbach’s alpha of 0.87 [17]. The validity of the Iranian version of the tool was confirmed through face and content validity, and its reliability was confirmed through Cronbach’s alpha coefficient [30]. Those participating in the study were given the opportunity to complete the questionnaires in a completely calm environment over a period of 30 min and were not rushed or disturbed in any way.

### Ethical approval

This study was approved by the Ethics Committee located in the Iranian University of Medical Sciences, Tehran, Iran (Number: IR.IUMS.REC. 1400.1083). In addition, informed written consent was obtained from the participants, who were fully informed of the purpose and procedures of the study. All participants included in this study were >18 years old as so did not require consent from a guardian/parent. Participants were also assured confidentiality in respect of their information. All methods were carried out in accordance with the study protocol, along with relevant guidelines and regulations associated with the Iranian University of Medical Sciences and professional regulatory bodies such as the Nursing and Midwifery Council.

### Analyses

The data were analyzed using SPSS V.24 (SPSS). Following assessment of skewness and kurtosis, the quantitative data were considered to be normally distributed. Descriptive statistics, including frequencies and percentages, mean and SD were used to understand demographic and variables associated with obstetric history, birth experience, maternal self-efficacy, and maternal performance. To compare the constructs of maternal performance and birth experience, the obtained scores were normalized to a maximum score of 100. To calculate each construct’s normalized score, each score was subtracted from the minimum score of that construct and divided by the difference of the maximum and minimum score of that construct. The final result obtained was then multiplied by 100.

To compare the total score of maternal performance, self-efficacy, and birth experience (quantitative variables) among low risk and high-risk pregnancies, an independent t- test was used. To compare the total score of maternal performance (quantitative variables) among demographic and variables associated with obstetric history (categorical variables), an independent t- test and ANOVA were used. The Pearson’s correlation coefficient test was used to determine the relationship between the total score of maternal performance with birth experience, maternal self-efficacy, demographic and variables associated with obstetric history that were considered quantitative variables.

To determine the relationship of each one of the independent variables (birth experience, maternal self-efficacy, demographic and variables associated with obstetric history) on the dependent variable (total score of maternal performance reported as quantitative variables), those variables that confirmed significance in the bivariate test (*p* < 0.05) were entered into a multiple linear regression model using a backward strategy. Prior to the multivariate analysis, regression assumptions, including normality of residuals, homogeneity of residual changes, and alignment of outliers and residuals independence, were examined, and confirmed. Results from the linear regression analysis are presented as beta coefficients with associated 95% CI. The level of statistical significance was set at *p* < 0.05.

## Results

The inclusion and exclusion criteria were examined in 765 potential participants. Many (*n* = 315) were excluded because they were not Iranian (*n* = 85), they gave birth via cesarean section (*n* = 175), they declined to consent to participation (*n* = 40), they had known psychological conditions or underlying physical conditions (e.g., lupus, heart disease, kidney disease) (*n* = 15). Of all eligible participants included in this study (*n* = 450), 49.6% were aged between 26–35 years old, 44.9% had a university education, 77.1% were housewives, and 54% had relatively favorable financial status. Of all births, 60.7% took place in hospitals, and 91.01% had only given birth once. Of all pregnancies, 87.6% were wanted and 73.1% were low risk. Most of the antenatal care (59.6%) had been given by gynecologists in the hospital clinic. The information related to the demographic characteristics and obstetric history of participants and the relationship of each of them with the total score of maternal performance are presented in Tables 1 and 2.

**Table 1:** Statistical indicators of maternal performance according to demographic variables in the participants

Variable		No	Percent	Mean	SD	*P* value
**Age (Year)**	25 ≥	200	44.44	19.90	12.685	0.078*
	26–35	223	49.56	91.22	12.343	
	36 ≤	27	6.00	95.89	10.020	
**Spouse’s age (Year)**	25 ≥	70	15.56	88.94	11.476	0.270*
	26–35	281	62.44	91.24	12.565	
	36 ≤	99	22.00	91/97	12.594	
**Level of education**	Primary school	35	7.78	91.03	13.809	*0.013
	Secondary School	60	13.33	86.73	11.505	
	Diploma	153	34.00	90.62	13.004	
	University education	202	44.89	92.64	11.715	
**Occupation**	Employed	103	22.89	93.60	11.953	** 0.017
	Housewife	347	77.11	90.28	12.468	
**Spouse’s level of education**	Primary school	25	5.56	83.56	14.157	* 0.020
	Secondary School	56	12.44	90.89	11.465	
	Diploma	169	37.56	91.66	12.414	
	University education	200	44.44	91.50	12.238	
**Spouse’s occupation**	Worker	82	18.22	87.62	12.144	* 0.022
	Employees	170	37.78	91.73	11.737	
	Self-employment	198	44.00	91.87	12.911	
**Ethnicity**	Turk	95	21.11	88.94	14.695	**0.005 ** *
	Kurdish	41	9.11	86.93	13.615	
	Lur	68	15.11	94.06	13.248	
	Fars	132	29.33	92.94	10.602	
	Other	114	25.33	90.28	10.628	
**Financial status**	Undesirable	68	15.11	89.16	17.008	0.067 *
	Fairly favorable	243	54.00	90.47	12.067	
	Optimal	139	30.89	92.96	9.994	

Significance level: *P* < 0.05

^*^One-way ANOVA, **Independent sample t-test

**Table 2:** Statistical indicators of maternal performance according to obstetric variables in the participants

Variable		No	Percent	Mean	SD	*P* value
**Place of birth**	Governmental hospital	273	60.67	89.74	12.907	0.006**
	Private of hospital	177	39.33	93.05	11.317	
**Number of previous births**	1	309	68.67	91.01	11.888	* **0.002**
	2	122	27.11	92.57	12.302	
	3 ≤	19	4.22	81.74	17.435	
**Number of abortions**	0	363	80.67	91.20	12.302	*0.259
	1	77	17.11	89.64	12.763	
	2 ≤	10	2.22	96.10	13.560	
**Gestational age at the time of birth (weeks)**	< 37	41	9.11	91.10	14.189	*0.394
	37- 39	297	66.00	90.52	12.091	
	40 – 41	112	24.89	92.40	12.60	
**Wanted pregnancy**	Yes	394	87.56	91.42	12.910	**0.084
	No	56	12.44	88.36	14.347	
**Pregnancy status**	Low risk	329	73.11	91.48	12.083	**0.218
	High risk	121	26.89	89.85	13.266	
**The reason for being high risk**	Diabetes	16	3.56	87.88	11.026	0.054*
	Anemia	5	1.11	87.60	9.633	
	Hypothyroidism	23	5.11	92.26	12.484	
	Preeclampsia	5	1.11	92	18.069	
	BMI > 29	8	1.78	84.50	13.649	
	Participant’s age > 35	12	2.67	97.25	8.368	
	Participant’s age < 18	6	1.33	75.17	8.280	
	Low birth weight	5	1.11	98	18.708	
	Preterm birth	41	9.11	89.32	13.787	
**Frequency of receiving antenatal care**	< 4	60	13.33	95.43	11.221	**0.001** *
	5 – 10	352	78.22	91.22	12.459	
	11 ≤	38	8.44	82.47	9.540	
**Place of antenatal care**	Hospital clinic	268	59.56	90.48	11.796	*0.055
	Midwife’s office	58	12.89	94.71	10.375	
	Health center	124	27.56	90.55	14.272	
**Provider of antenatal care**	Midwife	182	40.44	89.62	62.89	*0.125
	General physician	11	2.44	90.55	11.210	
	Obstetrician	257	57.11	92.07	12.073	
**Participation in childbirth preparation classes**	Yes	190	42.22	93.21	11.107	**0.001
	No	260	57.78	89.46	13.089	
**Epidural or spinal anesthesia separate**	Yes	148	32.89	90.10	13.133	**0.261
	No	302	67.11	91.50	12.047	
**Use of analgesics**	Yes	177	39.33	92.08	11.889	**0.154
	No	273	60.67	90.37	12.728	
**Birth suite**	Single Occupancy	256	56.89	92.56	12.124	**0.003
	Multiple Occupancy	194	43.11	89.04	12.547	
**Episiotomy**	Yes	343	76.22	90.88	12.250	**0.621
	No	107	23.78	91.56	12.988	
**Lead professional during birth**	Obstetrician	129	28.67	88.04	11.804	* **0.005**
	Midwife	108	24.00	92.11	13.692	
	Both	213	47.33	92.32	11.838	
Length of hospitalization in the birth suite*	≤ 4	122	27.11	94.47	12.277	* **0.–01**
	5—8	314	69.78	90.20	11.679	
	≥ 9	14	3.11	80.07	19.555	
Accompanying presence in the birth suite**	Yes	141	31.33	93.85	11.143	**0.001
	No	309	68.67	89.76	12.771	
Length of hospitalization (Day) *	1	372	82.67	90.69	12.085	* **0.012**
	2	66	14.67	94.32	13.412	
	3	12	2.67	84	13.518	
Hospitalization of the neonate after birth in the intensive care unit**	Yes	33	7.33	84.45	13.881	**0.001
	No	417	92.67	91.56	12.161	
Duration of hospitalization of the neonate in the intensive care unit*	0	417	92.67	91.56	12.161	* **0.006**
	1	13	2.89	88.54	8.263	
	2	9	2.00	81.22	14.948	
	3 ≤	11	2.44	82.27	17.878	

Significance level: *P* < 0.05

^*^One-way ANOVA, **Independent sample t-test

As presented in Table 3 and based on scores from 0 to 100, maternal competence had the highest mean score, and in relation to birth experience, professional support had the highest mean score.

**Table 3:** Mean and standard deviation of maternal performance, birth experience and maternal self-efficacy in participants

Total scores and domains	Max	Min	Mean	SD	Scores based on the 1–100				Scores based on the 1–4	
					**Max**	**Min**	**Mean**	**SD**	**Mean**	**SD**
**Maternal performance (0–120)**	118	51	91.04	12.418	98	43	75.87	10.348		
Domain 1: maternal needs ( 0–42)	42	9	30.58	6.272	100	21	72.81	14.933		
Domain 2: Maternal competence ( 0–78)	78	39	60.46	7.383	100	50	77.51	9.465		
**Childbirth experience (23–92)**	85	45	66.66	6..315	90	32	63.28	9.152	2.53	0.37
Domain 1: Participation ( 4–16)	16	6	11.57	1.823	100	17	63.06	15.193	2.52	0.61
Domain 2: Own capacity (8–32)	31	13	23.02	3.050	96	21	62.60	12.708	2.5	0.51
Domain 3: Professional support (5–20)	20	8	15.64	2.055	100	20	70.92	13.699	2.83	0.55
Domain 4: perceived safety (6–24)	22	10	16.44	1.924	89	22	57.98	10.691	2.32	0.43
**Maternal self-efficacy ( 10–40)**	40	22	32.56	3.452	100	40	75.20	11.505		

Among all participants (*n* = 450), 121 (26.9%) had a high-risk pregnancy (HRP) and 329 (73.1%) had a low-risk pregnancy (LRP). In the comparison of the mean score of birth experience, self-efficacy and maternal performance in two groups with high-risk and low-risk pregnancies, a statistically significant difference was only seen in the score relating to birth experience (mean (SD) of childbirth experience of LRP and HRP 62.02 (9.92) and 60.5 (10.42), respectively) (*p* = 0.01). In the comparison of the other two variables, self-efficacy and maternal performance, this difference between the two HRP and LRP groups were not statistically significant (mean (SD) of maternal performance of LRP and HRP 88.16 (11.14) and 87.16 (12.46), respectively; mean (SD) of self-efficacy of LRP and HRP 32.5 (3.32) and 32.7 (3.8), respectively).

We identified statistically significant correlations between maternal performance and domains related to birth experience (r = 0.52, *P* < 0.001), participation (r = 0.46, *P* < 0.001), own capacity (r = 0.43, *P* < 0.001), professional support (r = 0.35, *P* < 0.001) and perceived safety (r = 0.2, *P* < 0.001). Evidently, these factors improve maternal performance. Moreover, maternal self-efficacy had a statistically significant relationship with maternal performance (r = 0.56, *P* < 0.001).

To estimate the impact of each of the demographic and obstetric variables, birth experience and maternal self-efficacy on maternal performance and to explain the changes, all the variables that had *p* < 0.05 based on the results of Tables 1 and 2 were entered into the multiple linear regression model using a Backward strategy. Among the variables included in the model, the variables of birth experience, maternal self-efficacy’ spouse's occupation, number of births, frequency of receiving antenatal care, length of stay in the birth suite, and length of stay in the hospital remained in the model. As the results presented in Table 4 demonstrate, participants whose spouses were workers had a higher maternal performance by 5.78 units and participants whose spouses were employees were higher by 3.99 units than those whose spouses were self-employed. Participants who had two births had a lower maternal performance score of 8.46 units compared to those who had three or more previous births. Participants who received antenatal care 5–10 times, compared to those who received antenatal care more than 11 times, had a lower maternal performance score of 6.68 units. Participants whose length of hospitalization in the birth suite was less than 4 h, compared to participants who were hospitalized in the birth suite for more than 9 h had a lower maternal performance score of 2.22 points. Participants who were hospitalized for one day had a higher maternal performance score of 2.84 points compared to participants who were hospitalized for three days. Overall, 53.2% of the changes in the variable of ‘maternal performance’ are caused by independent variables, birth experience, maternal self-efficacy’ spouse's occupation, number of births, frequency of receiving antenatal care, length of hospitalization in the birth suite and length of hospitalization in the hospital is justified.

**Table 4:** Results of multiple linear regression analysis investigating the effect of demographic and obstetric variables, birth experience and maternal self-efficacy on maternal performance

**Independent variables**		**Unstandardized coefficients B**	**Standardized coefficient beta**		**95% CI for B**	***P*** **-value**	**R2**
**Childbirth experience**		0.63	0.320		0.46 to 0.79	0.001	0.532
**Maternal self-efficacy**		1.53	o.424		1.25 to 1.80	0.070	
**Level of education**	**Primary school**	-3.75	-0.103		-7.81 to 0.30	0.065	
	**Secondary school**	-3.67	0.140		-7.57 to 0.23	**0.065**	
	**Diploma**	-2.92	-0.117		-7.20 to 1.35	0.180	
	**University education**	Reference category					
**Occupation**	Housewife	1.3	0.044		-0.92 to 3.51	0.215	
	Employed	Reference category					
**Spouse's level of education**	**Primary school**	0.97	0.026		-3.54 to 5.48	0.673	
	**Secondary school**	-0.204	-0.008		-4.56 to 4.15	0.927	
	**Diploma**	-1.89	-0.076		-6.67 to 2.88	0.437	
	**University education**	Reference category					
**Spouse's occupation**	Worker	5.78	0.226		2.78 to 8.77	0.001	
	Employees	3.99	0.160		1.31 to 6.66	0.004	
	Self-employment			Reference category			
**Ethnicity**	Turk	-1.11	-0.026		-4.62 to 2.39	0.534	
	Kurdish	2.27	0.066		-0.66 to 5.21	0.129	
	Lur	1.72	0.063		-0.77 to 4.21	0.175	
	Fars	-0.023	-0.001		-2.73 to 2.68	0.987	
	Other			Reference category			
**Place of childbirth**	Governmental hospital	-1.7	-0.067		-3.91 to 0.52	0.133	
	Private of hospital	Reference category					
**Number of childbirth**	1	-1.012		- 0.036	-2.99 to 0.97	0.316	
	2	-8.46		-0.137	-12.86 to 4.06	0.001	
	3 ≤	Reference category					
**Receiving antenatal care**	< 4	-0.97		0.032	-3.65 to 1.71	0.477	
	5—10	-6.68		-0.150	-10.82 to 2.52	0.002	
	11 ≤	Reference category					
**Place of antenatal care**	Hospital clinic	-0.12		0.003	-2.79 to 2.56	0.933	
	Midwife's office	-0.105		0.004	-2.23 to 2.02	0.923	
	Health center	Reference category					
**Birth suite**	Single Occupancy	1.502		0.060	-0.55 to 3.55	0.151	
	Multiple Occupancy	Reference category					
**Lead professional during birth**	Obstetrician	2.62		0.090	-0.01 to 5.24	0.050	
	Midwife	1.98		0.080	-0.16 to 4.12	0.071	
	Both	Reference category					
**Length of hospitalization in the birth suite**	≤ 4	-2.22		-0.082	-4.21 to 0.22	0.029	
	5—8	-3.76		-0.053	-9.25 to 1.73	0.180	
	≥ 9	Reference category					
**Accompanying presence in the birth suite**	Yes	-1.05		-0.039	-3.07 to 0.97	0.307	
	No			Reference category			
**Length of hospitalization (Day)**	1	2.84		0.081	0.31 to 5.37	0.028	
	2	4.08		0.053	-1.52 to 9.68	0.153	
	3	Reference category					
**Hospitalization of the neonate after birth in the intensive care unit**	Yes	-1.34		0.031	-2.52 to 1.89	0.298	
	No	Reference category					
**Duration of hospitalization of the neonate in the intensive care unit**	0	1.74		0.023	-3.43 to 6.90	0.509	
	1	0.04		0.001	-6.12 to 6.20	0.990	
	2	-4.14		-0.052	-9.83	1.54	
	3 ≤	-		Reference category			

## Discussion

This study aimed to determine maternal performance after childbirth and its predictors. Mean scores related to maternal performance, maternal needs, and maternal competence were found to be 91.04, 30.58, and 60.46, respectively. The mean scores related to maternal performance in several other studies conducted in Tabriz (Iran) have been reported to be far higher than this at 97.4, 93.3 and 97.4, respectively [21, 31, 32]. These higher scores suggest that participants had a better experience in Tabriz than participants in our study did. When compared, the evidence points to geographical areas where future efforts should be focused.

A separate study reported a lower maternal performance score of 80 in those with a positive screen for depression [25], and in a later study, the mean maternal performance score was reportedly lower again at 81.5 [26]. In these two compared studies, participants had experienced depression during pregnancy. Yet in the present study participants self-reported having no mental health issues. This is significant, as maternal performance is known to increase where the severity of depression is reduced and overall mental health is improved [26, 33]. In the present study, the highest score related to the domain of ‘maternal competence’ was similar to results reported elsewhere in Iran [34]. Yet in two other studies which alternatively used the IFSAC tool, the domain of ‘neonatal care’ and ‘self-care’ had the highest mean score, and social activity had the lowest mean score [19, 35]. Future research could usefully investigate why these differences may occur when differing data collection tools are used and bring uniformity to studies using larger sample sizes.

The mean score of birth experience was 2.53 (the range being 1–4). As higher scores indicate a more positive childbirth experience, this score indicates opportunities for improvement in the experiences of childbirth in Iran. A comparison of scores demonstrated ‘professional support’ had the highest mean score and ‘perceived safety’ had the lowest mean score. Vahidi et al. who similarly used the CEQ2, obtained a birth experience score of 2.7 (1–4), whilst ‘professional support’ and ‘perceived safety’ had the highest and lowest mean scores respectively [36]. The results of this study are similar to the present study in terms of the birth experience score and the highest and lowest score. In another study, the mean score of birth experience was reported at 2.71 (1–4) whilst ‘professional support’ and ‘personal capacity’ scored the highest and lowest mean scores respectively [27]. This highest score aligns with those reported in the present study. Similarly, Khalife-Ghaderi reported the mean score of birth experience to be 55.73%, where ‘professional support’ and ‘participation’ obtained the highest and lowest mean scores respectively. These results are also consistent in terms of high ‘professional support’ [37]. Yet in results reported elsewhere, the domain of ‘personal capacity’ and ‘professional support’ obtained the highest and lowest mean scores, respectively [38]. This is inconsistent with the results presented here, yet only 19% of participants in this earlier study gave birth in a private hospital, whereas in the current study, approximately 33% gave birth in a private hospital, and thus likely received supplementary care and support. This may somewhat explain the differences apparent within the domains, and future qualitative research could usefully explore some of the more nuanced reasons behind such differences.

The mean score of maternal self-efficacy in the present study was 32.56, which includes 75% of the total score. In another study, the mean score of self-efficacy was 65.58, which is equivalent to 72% of the total score available [39], and close to the score reported in the present study. Mirghafourvand and Bagherinia also reported that the mean self-efficacy score in their study was 33.8, equivalent to 84% of the total score [40]. Elsewhere, the parent expectations survey (PES) was used to measure maternal self-efficacy, and the mean score of maternal self-efficacy was again close to the results of our study [7]. Yet in another study conducted with Italian participants, the mean score of self-efficacy was 37% of the total score [41]. Yet in this Italian context, many participants gave birth prematurely and their babies were hospitalized in the neonatal intensive care unit (NICU). It is possible that this factor caused low self-efficacy in this particular sample, particularly as evidence suggests that performing specialized medical services in the NICU reduces self-efficacy of participants with premature babies overall [42].

In the current study there was no statistically significant difference identified in maternal performance scores between the two groups with high-risk and low-risk pregnancies. Similarly, Vahidi et al. reported in another cross-sectional study that there was no statistically significant difference in terms of maternal performance score between groups with complicated pregnancies (diabetes, preeclampsia, anemia, and hypothyroidism) and those without complications. Yet in this earlier study, the Barkin Index of Maternal Functioning (BIMF) tool was used to measure maternal functioning [36]. Nevertheless, these findings indicate that maternal performance may not be markedly affected by the level of risk associated with pregnancy. This is important because postpartum maternal functioning can significantly influence the establishment and maintenance of a successful bonding relationships, and a high level of maternal functioning is likely to be associated with positive infant developmental outcomes. Indeed, other studies have identified that; number of births, birth experience, type of birth, and maternal and neonatal complications influence postpartum maternal performance in the general population [23, 43]. Moreover, parents whose babies were admitted to the intensive care unit have been found to have a higher adaptation score with the maternal role, and those who had full-term babies have been found to have a higher adaptation score with the maternal role compared to mothers who had preterm babies [44]. Such differences in scores indicate a need for further qualitative research in this area to understand the mechanisms behind this in greater detail.

Numerous other variables including maternal age, perception of the childbirth experience, preterm childbirth, social stress, social support, and personality traits in the formation of maternal identity and functioning have been found to affect maternal adjustment, including social and psychological factors [43–45].Those with high-risk pregnancies may experience multiple hospitalizations during pregnancy, which may also lead people to experience different emotions [45]. Thus in the long term, those who experience high-risk pregnancies may experience depression or PTSD. This may in turn disrupt the acquisition of a parenting role and impact upon infant bonding and thriving in this context.

In the present study, there was statistically significant difference identified in childbirth experience scores between those with high-risk and low-risk pregnancies in that those who had a high-risk pregnancy had a lower childbirth experience score. Tabaghdehi et al. reported the factors that lead to high-risk pregnancy and childbirth which increase maternal stress and anxiety and how these can lead to a negative birth experience [7]. The admission of a newborn in the neonatal unit, a low Apgar score after birth can also cause concern and lead to a negative experience of birth [46]. Moreover, during preterm labor, birthing parents express "fear of losing the baby" due to the early onset of labor [47]. "Fear of losing a baby" may be experienced as a "threat" in the psychological sense [48]. This sense of threat can ignite the perception again of a traumatic birth experience [49]. Nevertheless, Najafi et al. report no statistically significant difference in terms of the birth experience score between those with those full-term and pre-term babies [49]. Again, further qualitative research in this area could unearth the reasons behind such experiences for greater nuance.

Based on the results of the present study, there was no statistically significant difference identified in self-efficacy scores between those with high-risk and low-risk pregnancies. A separate study determined depression, anxiety, self-efficacy, and self-esteem among those with high-risk pregnancies who experienced adverse outcomes in previous pregnancies. Results demonstrated that this group had a higher level of depression compared to the low-risk group, but surprisingly, they had a higher level of self-efficacy and self-esteem during pregnancy and after pregnancy [50]. Chronic diseases also have a significant and well-known relationship with the development of pregnancy anxiety. Those pregnant who suffer from chronic diseases usually face many negative effects caused by their mental ill health [51]. These people experience more anxiety and thus their self-efficacy may be lower due to anxiety [52, 53]. This is important because based on Bandura’s theory [54], anxiety symptoms could be regulated and triggered by higher perceived levels of self-efficacy. Overall, future research could usefully explore how mental health may be improved in these populations with regard to their childbirth experience, and with larger sample sizes.

In the present study, birth experience and its dimensions had a statistically significant correlation with maternal performance. Evidently, by improving the birth experience, maternal performance improves in turn. Indeed, elsewhere there has been found a positive and significant relationship between maternal performance and birth experience [21] consistent with the present study. Other evidence demonstrates that participants who experienced labor complications during childbirth had weaker maternal performance one month after [55]. Also, several factors including anxiety and depression in the postpartum period can be related to factors such as the experience of childbirth and the conditions of caring for the neonate which cause disruption in the adaptation of participants in the postpartum period, and even the necessary measures to disrupt their health [56]. In this latter study, a relationship was also found between birth experience and maternal performance. Thus, it remains key to enhance birth experience in order reap the rewards of enhanced maternal performance.

We report a significant statistical relationship found between maternal self-efficacy and maternal performance. Self-efficacy is an important indicator of a successful role transition and an important predictor of maternal infant care behavior [29]. Ultimately, a participant with high self-efficacy may be more successful in establishing communication and warm attachment with the neonate, while low maternal self-efficacy is related to low attachment and vulnerability [57]. In general, self-efficacy is defined as a parent's beliefs or judgments about the ability to organize and execute a set of parenting tasks [58]. Playing a strong maternal role creates a sense of competence and satisfaction, secure attachment, as well as cultivating responsible behavior in parents, and facilitating the growth and development of the neonate [59]. Thus strategies which enhance self-efficacy in new parents should be encouraged.

Regarding the relationship between demographic variables and maternal performance, there was a statistically significant relationship between participant's education level, participant's employment status, spouse's education level, spouse's employment status, and ethnicity. Maternal performance had no significant relationship with participant's age, spouse's age and financial status. Consistent with these results, Mirghafourvand et al., identified that the participant's education and spouse's occupation also have a significant direct relationship with maternal performance [19]. In the present study, participants who had a higher level of education performed better than participants who had only primary education. In another study, participants with less education also demonstrated poorer performance [46], and in two other studies, the level of education had a positive and significant relationship with maternal performance, where those who had a higher level of education demonstrated enhanced maternal performance [31, 33]. In other studies, there was a significant relationship identified between participant occupation and maternal performance [34]. Whilst the results of some studies in relation to the participant’s job are consistent with the results of the present study, in another study, the participant's maternal performance decreased in line with their income and educational level [60]. This is not consistent with the results presented here. Nevertheless, in Lax's study, participants were infected by COVID-19, and perceived stress caused participants who had a higher level of education and income to feel more inadequate in maternal competence [60]. Overall, it will be important to ensure that levels of both education and employment are high prior to pregnancy and childbirth taking place in these populations to improve performance overall.

In one study, participant's age and spouse's age were related [36]. Barkin et al. concluded that maternal age has a significant and inverse relationship with maternal performance, and that maternal performance becomes weaker as the participant's age increases [25]. Perhaps the reason for this difference is that in Barkin's study, the participants all depression or mental health problems. In the present study, financial status was not related to performance. In contrast to this, the adequacy of family income and household financial level has been found to have a direct and significant relationship with maternal performance elsewhere [21]. Yet in this study, 364 out of the 483 participants had relatively favorable financial status to begin with, and only 74 individuals had insufficient income. This homogeneity in financial status may somewhat explain this difference and prompt the need for more diverse samples in future. Again, in contrast to the results presented here, another study reported that the financial level of the household had a direct and significant relationship with maternal performance [19]. In future it will be important to explore these inconsistencies in the evidence, yet higher incomes may afford participants less stress and increased opportunities in domains such as health and education which in turn may lead to enhanced maternal support overall.

We identified statistically significant relationships between maternal performance and place of childbirth, number of previous births, frequency of receiving antenatal care, participation in birth preparation classes, childbirth room, birth agent, length of time in the birth suite, the presence of a companion in the birth suite, the duration of hospital stay, the hospitalization of the newborn in the intensive care unit after birth, and the duration of the newborn's hospitalization in the intensive care unit. There were no significant statistical correlations with other variables. Elsewhere, there has been no significant relationship found between variables of pregnancy desirability and maternal performance [31]. This is consistent with the results presented here. Equally consistent is the finding that maternal performance of participants who received antenatal care was higher than participants who did not receive antenatal care [61]. Also, the number of births has previously been found to have a direct and significant relationship with maternal performance, so that multiparous participants performed better than primiparous participants [19]. These results are not surprising yet confirm once again the importance of antenatal education and deeper understandings in relation to how childbirth is experienced in hospital settings.

## Limitations and suggestions for future research

A clear limitation in this study was that only participants from the west and north-west regions of Tehran were included. Moreover, due to the design of this study, those who experienced birth via cesarean section were also excluded. Considering that not all participants with a high-risk pregnancy and/or birth were able to enter the study in this study, results cannot be generalized to all high-risk pregnancies and births. Future research is required in other geographical areas of Tehran, examining maternal performance in those who have given birth via cesarean section and those who experience high-risk pregnancy and childbirth. In the present study, maternal performance was investigated in those who reported having good mental health. Future research could usefully compare maternal performance in those with and without reportedly good mental health.

## Conclusion

Ultimately, birth experience and self-efficacy with maternal performance have a significant relationship with one another. Thus, applying strategies to improve the experience of childbirth and self-efficacy would result in improved maternal performance in the postpartum period. Enhanced antenatal care aimed at improving maternal function after giving birth will be crucial in this pursuit. Moreover, in realizing that the duration of hospitalization in the birth suite and the hospital in general are predictors of maternal performance, it will be necessary to provide highly compassionate care and robust evidence-based education following childbirth. Promoting evidence-based, person-centered, and respectful care during pregnancy and birth are required to optimise all outcomes in this regard.

## Data Availability

The datasets generated and analyzed during the current study are not publicly available due to the confidentiality of information, but they can be available through the corresponding author on reasonable request.

## References

[1] Fenaroli V, Molgora S, Dodaro S, Svelato A, Gesi L, Molidoro G, Saita E, Ragusa A (2019). The childbirth experience: obstetric and psychological predictors in Italian primiparous women. BMC Pregnancy Childbirth.

[2] Thomson G, Garrett C (2019). Afterbirth support provision for women following a traumatic/distressing birth: survey of NHS hospital trusts in England. Midwifery.

[3] Gharaei T, Amiri-Farahani L, Haghani S, Hasanpoor-Azghady SB (2020). The effect of breastfeeding education with grandmothers’ attendance on breastfeeding self-efficacy and infant feeding pattern in Iranian primiparous women: a quasi-experimental pilot study. Int Breastfeed J.

[4] Abdollahpour S, Mousavi SA, Motaghi Z, Keramat A, Khosravi A (2017). Prevalence and risk factors for developing traumatic childbirth in Iran. J Public Health.

[5] World Health Organization. WHO recommendations on intrapartum care for a positive childbirth experience. World Health Organization; 2018. 30070803

[6] Oyetunji A, Chandra P (2020). Postpartum stress and infant outcome: A review of current literature. Psychiatry Res.

[7] Hosseini Tabaghdehi M, Kolahdozan S, Keramat A, Shahhossein Z, Moosazadeh M, Motaghi Z (2020). Prevalence and factors affecting the negative childbirth experiences: a systematic review. J Matern Fetal Neonatal Med.

[8] McLachlan HL, Forster DA, Davey MA, Farrell T, Flood M, Shafiei T, Waldenström U (2016). The effect of primary midwife-led care on women's experience of childbirth: results from the COSMOS randomised controlled trial. BJOG: Int J Obstetr Gynaecol..

[9] Hajizadeh K, Vaezi M, Meedya S, Mohammad Alizadeh Charandabi S, Mirghafourvand M (2020). Respectful maternity care and its relationship with childbirth experience in Iranian women: a prospective cohort study. BMC Pregnancy Childbirth..

[10] Hajizadeh K, Vaezi M, Meedya S, Mohammad Alizadeh Charandabi S, Mirghafourvand M (2020). Prevalence and predictors of perceived disrespectful maternity care in postpartum Iranian women: a cross-sectional study. BMC Pregnancy Childbirth..

[11] Sedigh Mobarakabadi S, Mirzaei Najmabadi K, Ghazi TM (2015). Ambivalence towards childbirth in a medicalized context: a qualitative inquiry among Iranian mothers. Iran Red Crescent Med J.

[12] Amiri-Farahani L, Gharacheh M, Sadeghzadeh N, Peyravi H, Pezaro S (2022). Iranian midwives’ lived experiences of providing continuous midwife-led intrapartum care: a qualitative study. BMC Pregnancy Childbirth.

[13] Ardakani ZB, Navabakhsh M, Ranjbar F, Tremayne S, Akhondi MM, Tabrizi AM (2020). Dramatic rise in cesarean birth in Iran: a coalition of private medical practices and womenâ s choices. Int J Women's Health Reprod Sci.

[14] Kazemi S, Dencker A, Pazandeh F, Montazeri A, Sedigh-Mobarakabadi S, Hajian S (2020). Psychometric evaluation of the Persian version of the childbirth experience questionnaire (CEQ). Biomed Res Int.

[15] Dencker A, Taft C, Bergqvist L, Lilja H, Berg M (2010). Childbirth experience questionnaire (CEQ): development and evaluation of a multidimensional instrument. BMC Pregnancy Childbirth.

[16] Aydın R, Kukulu K (2018). Adaptation of the Barkin scale of maternal functioning and examination of the psychometric properties. Health Care Women Int.

[17] Barkin JL, Wisner KL, Wisniewski SR (2014). The psychometric properties of the Barkin index of maternal functioning. J Obstet Gynecol Neonatal Nurs.

[18] Jones E, Stewart F, Taylor B, Davis PG, Brown SJ. Early postnatal discharge from hospital for healthy mothers and term infants. Cochr Database Syst Rev. 2021(6). 10.1002/14651858.CD002958.pub2 PMC8185906 34100558

[19] Mirghafourvand M, Mohammad Alizadeh Charandabi S, Fathi F, Razzag S (2021). Predictors of maternal functional status during postpartum period. Hayat..

[20] Albanese AM, Geller PA, Steinkamp JM, Barkin JL (2020). In their own words: A qualitative investigation of the factors influencing maternal postpartum functioning in the united states. Int J Environ Res Public Health.

[21] Havizari S, Ghanbari-Homaie S, Eyvazzadeh O, Mirghafourvand M (2022). Childbirth experience, maternal functioning and mental health: how are they related?. J Reprod Infant Psychol.

[22] Bell AF, Rubin LH, Davis JM, Golding J, Adejumo OA, Carter CS (2019). The birth experience and subsequent maternal caregiving attitudes and behavior: a birth cohort study. Arch Womens Ment Health.

[23] Fathi F, Mohammad-Alizadeh-Charandabi S, Mirghafourvand M (2018). Maternal self-efficacy, postpartum depression, and their relationship with functional status in Iranian mothers. Women Health..

[24] Salonen AH, Kaunonen M, Åstedt-Kurki P, Järvenpää AL, Isoaho H, Tarkka MT (2019). Parenting self-efficacy after childbirth. J Adv Nurs.

[25] Barkin JL, Wisner KL, Bromberger JT, Beach SR, Wisniewski SR (2016). Factors associated with postpartum maternal functioning in women with positive screens for depression. J Womens Health.

[26] Barkin JL, Beals L, Bridges CC, Ezeamama A, Serati M, Buoli M, Erickson A, Chapman M, Bloch JR (2021). Maternal functioning and depression scores improve significantly with participation in visiting moms® program. J Am Psychiatr Nurses Assoc.

[27] Ghanbari-Homayi S, Dencker A, Fardiazar Z, Jafarabadi MA, Mohammad-Alizadeh-Charandabi S, Meedya S, Mohammadi E, Mirghafourvand M (2019). Validation of the Iranian version of the childbirth experience questionnaire 2.0. BMC Pregnancy Childbirth..

[28] Teti DM, Gelfand DM (1991). Behavioral competence among mothers of infants in the first year: The mediational role of maternal self-efficacy. Child Dev.

[29] Mirghafourvand M, Mohammad-Alizadeh-Charandabi S, Asghari Jafarabadi M, Fathi F (2016). Psychometric properties of maternal self-efficacy questionnaire in a population of Iranian mothers. J Child Fam Stud..

[30] Mirghafourvand M, Barkin JL, Jafarabadi MA, Karami F, Ghanbari-Homayi S (2019). The psychometric properties of the Barkin index of maternal functioning (BIMF) for the Iranian population. BMC Womens Health.

[31] Gholizadeh Shamasbi S, Barkin JL, Ghanbari-Homayi S, Eyvazzadeh O, Mirghafourvand M (2020). The relationship between maternal functioning and mental health after childbirth in Iranian women. Int J Environ Res Public Health.

[32] Karami Chamgurdani F, Barkin JL, Curry CL, Mirghafourvand M (2020). Comparison of maternal functioning between Iranian mothers with and without depressive symptoms: a case-control study. Int J Environ Res Public Health.

[33] Floyd James K, Smith BE, Robinson MN, Thomas Tobin CS, Bulles KF, Barkin JL (2023). Factors associated with postpartum maternal functioning in black women: a secondary analysis. J Clin Med.

[34] Ahmadpour P, Curry C, Jahanfar S, Nikanfar R, Mirghafourvand M (2023). Family and spousal support are associated with higher levels of maternal functioning in a study of Iranian postpartum women. J Clin Med.

[35] Mirghafourvand M, Mohammad-Alizadeh-Charandabi S, Jafarabadi MA, Soltanpour S, Aghamiri V, Bagherinia M. Physical activity during pregnancy and its relationship with the functional status of primiparous women six weeks after childbirth: a cohort study. J Clin Diagnost Res. 2018;12(11).

[36] Vahidi F, Mirghafourvand M, Naseri E, Ghanbari-Homaie S (2023). Birth-related posttraumatic stress disorder and negative childbirth experience related to maternal functioning among adolescent mothers: a cross-sectional study. BMC Pregnancy Childbirth.

[37] Khalife-Ghaderi F, Amiri-Farahani L, Haghani S, Hasanpoor-Azghady SB (2021). Examining the experience of childbirth and its predictors among women who have recently given birth. Nurs Open.

[38] Azarkish M, Malakouti J, Mirghafourvand M (2022). Relationship between childbirth experience and sexual function and sleep quality in Iranian postpartum women: a cross-sectional study. J Psychosoc Nurs Ment Health Serv.

[39] Sari C, Altay N (2020). Effects of providing nursing care with web-based program on maternal self-efficacy and infant health. Public Health Nurs.

[40] Mirghafourvand M, Bagherinia M (2018). Relationship between maternal self-efficacy and functional status four months after delivery in Iranian primiparous women. J Psychosom Obstet Gynecol.

[41] Pedrini L, Ferrari C, Ghilardi A (2019). Psychometric properties of the Italian perceived maternal parenting self-efficacy (PMP SE). J Clin Psychol Med Settings.

[42] Brockway M, Benzies KM, Carr E, Aziz K (2018). Breastfeeding self-efficacy and breastmilk feeding for moderate and late preterm infants in the Family Integrated Care trial: a mixed methods protocol. Int Breastfeed J.

[43] Schobinger E, Vanetti M, Ramelet AS, Horsch A (2022). Social support needs of first-time parents in the early-postpartum period: A qualitative study. Front Psych.

[44] Ahmadpour P, Jahanfar S, Hamed Bieyabanie M, Mirghafourvand‬‬‬‬‬‬‬‬‬‬‬‬‬‬ M. Predictors of maternal role adaptation in Iranian women: a cross-sectional study. BMC Pregnancy Childbirth. 2022;22(1):367. 10.1186/s12884-022-04702-2 PMC9047459 35484515

[45] Rai S, Pathak A, Sharma I (2015). Postpartum psychiatric disorders: Early diagnosis and management. Indian J Psychiatry.

[46] Wigert H, Nilsson C, Dencker A, Begley C, Jangsten E, Sparud-Lundin C, Mollberg M, Patel H (2020). Women’s experiences of fear of childbirth: a metasynthesis of qualitative studies. Int J Qual Stud Health Well Being.

[47] Di Chiara M, Laccetta G, Gangi S, De Santis B, Spiriti C, Attenni M, Bertolaso L, Boscarino G, De Nardo MC, Ciambra G, Parisi P, Terrin G (2022). Risk factors and preventive strategies for post-traumatic stress disorder in neonatal intensive care unit. Front Psychol.

[48] Alfaro Blazquez R, Ferrer Ferrandiz E, Gea Caballero V, Corchon S, JuarezVela R (2019). Women’s satisfaction with maternity care during preterm birth. Birth.

[49] Najafi Z, Mirghafourvand M, Ghanbari-Homaie S (2023). Are women with preterm labour at risk for negative birth experience? a comparative cross-sectional study from Iran. BMC Pregnancy Childbirth.

[50] Scabia A, Donati MA, Primi C, Lunardi C, Lino G, Dèttore D, Vannuccini S, Mecacci F. Depression, anxiety, self-efficacy and self-esteem in high risk pregnancy. Minerva Obstet Gynecol. 2022. 10.23736/S2724-606X.22.05116-8. Epub ahead of print. PMID: 35829625. 10.23736/S2724-606X.22.05116-8 35829625

[51] Ankumah NE, Sibai BM (2017). Chronic hypertension in pregnancy: diagnosis, management, and outcomes. Clin Obstet Gynecol.

[52] Tang X, Lu Z, Hu DH, Zhong XN (2019). Influencing factors for prenatal Stress, anxiety and depression in early pregnancy among women in Chongqing. China J Affect Disord.

[53] Hou QZ, Li SS, Jiang C, Huang YL, Huang LL, Ye J (2018). The associations between maternal lifestyles and antenatal stress and anxiety in Chinese pregnant women: a cross-sectional study. Sci Rep.

[54] Colleen JH (1988). Social foundations of thought and action: a social cognitive theory. Behav Chang.

[55] Norhayati MN, Hazlina NH, Aniza AA (2016). Functional status of women with and without severe maternal morbidity: a prospective cohort study. Women and Birth.

[56] Bagherinia M, Mirghafourvand M, Shafaie FS (2017). The effect of educational package on functional status and maternal self-confidence of primiparous women in postpartum period: a randomized controlled clinical trial. J Matern Fetal Neonatal Med.

[57] Ghannad Z, Aalipor, Baranian (2019). Investigating the role of self-care dimensions in maternal self-efficacy of women referred to health centers in Ahvaz. Women Fam Stud.

[58] Khushk A, Dacholfany MI, Abdurohim D, Aman N (2023). Social learning theory in clinical setting: Connectivism, constructivism, and role modeling approach. Health Econ Manage Rev.

[59] Bagherinia M, Meedya S, Mirghafourvand M (2018). Association between maternal sense of competence and self-efficacy in primiparous women during postpartum period. Shiraz E-Med J.

[60] Lax ES, Novak SA, Webster GD (2023). Maternal functioning and psychological distress during the COVID-19 pandemic. J Womens Health.

[61] Cresswell JA, Barbour KD, Chou D, McCaw-Binns A, Filippi V, Cecatti JG, Barreix M, Petzold M, Kostanjsek N, Cottler-Casanova S, Say L (2020). Measurement of maternal functioning during pregnancy and postpartum: findings from the cross-sectional WHO pilot study in Jamaica, Kenya, and Malawi. BMC Pregnancy Childbirth.

